# Perspective for Precision Medicine for Tuberculosis

**DOI:** 10.3389/fimmu.2020.566608

**Published:** 2020-10-08

**Authors:** Christoph Lange, Rob Aarnoutse, Dumitru Chesov, Reinout van Crevel, Stephen H. Gillespie, Hans-Peter Grobbel, Barbara Kalsdorf, Irina Kontsevaya, Arjan van Laarhoven, Tomoki Nishiguchi, Anna Mandalakas, Matthias Merker, Stefan Niemann, Niklas Köhler, Jan Heyckendorf, Maja Reimann, Morten Ruhwald, Patricia Sanchez-Carballo, Dominik Schwudke, Franziska Waldow, Andrew R. DiNardo

**Affiliations:** ^1^Division of Clinical Infectious Diseases, Research Center Borstel, Borstel, Germany; ^2^German Center for Infection Research (DZIF) Partner Site Borstel-Hamburg-Lübeck-Riems, Borstel, Germany; ^3^Respiratory Medicine and International Health, University of Lübeck, Lübeck, Germany; ^4^Cluster of Excellence Precision Medicine in Chronic Inflammation, Kiel, Germany; ^5^Department of Medicine, Karolinska Institute, Stockholm, Sweden; ^6^Department of Internal Medicine, Radboud Center of Infectious Diseases (RCI), Radboud University Medical Center, Nijmegen, Netherlands; ^7^Department of Pulmonology and Allergology, Nicolae Testemitanu University of Medicine and Pharmacy, Chisinau, Moldova; ^8^School of Medicine, University of St Andrews, St Andrews, United Kingdom; ^9^The Global Tuberculosis Program, Texas Children's Hospital, Immigrant and Global Health, Department of Pediatrics, Baylor College of Medicine, Houston, TX, United States; ^10^Molecular and Experimental Mycobacteriology, Research Center Borstel, Borstel, Germany; ^11^Foundation of Innovative New Diagnostics (FIND), Geneva, Switzerland; ^12^Bioanalytical Chemistry, Priority Area Infection, Research Center Borstel, Leibniz Lung Center, Borstel, Germany; ^13^Airway Research Center North, German Center for Lung Research (DZL), Borstel, Germany

**Keywords:** precision medicine, tuberculosis, tailor-made regimen, mycobacterial genotypes, endotypes

## Abstract

Tuberculosis is a bacterial infectious disease that is mainly transmitted from human to human via infectious aerosols. Currently, tuberculosis is the leading cause of death by an infectious disease world-wide. In the past decade, the number of patients affected by tuberculosis has increased by ~20 percent and the emergence of drug-resistant strains of *Mycobacterium tuberculosis* challenges the goal of elimination of tuberculosis in the near future. For the last 50 years, management of patients with tuberculosis has followed a standardized management approach. This standardization neglects the variation in human susceptibility to infection, immune response, the pharmacokinetics of drugs, and the individual duration of treatment needed to achieve relapse-free cure. Here we propose a package of precision medicine-guided therapies that has the prospect to drive clinical management decisions, based on both host immunity and *M. tuberculosis* strains genetics. Recently, important scientific discoveries and technological advances have been achieved that provide a perspective for individualized rather than standardized management of patients with tuberculosis. For the individual selection of best medicines and host-directed therapies, personalized drug dosing, and treatment durations, physicians treating patients with tuberculosis will be able to rely on these advances in systems biology and to apply them at the bedside.

## Introduction

Tuberculosis is the leading cause of death attributed to a single microbial pathogen world-wide (1). The World Health Organization (WHO) estimates, that in the year 2018 globally 10 million people newly developed tuberculosis and 1.5 million people died from this disease (1). Despite the enormous burden of tuberculosis on healthcare systems, especially in developing countries, research on new preventive and diagnostic methods and novel therapies against tuberculosis has gained new momentum only recently.

For the past five decades, patients with tuberculosis have received the same standard therapies without acknowledging differences in human immunity, pharmacokinetics or variations in the pathogenesis or of the causative microbe, *Mycobacterium tuberculosis*. Treatment was empiric or adopted to results of phenotypic drug-susceptibility testing that takes several weeks to months to become available. Patient-tailoring was limited to adjusting dosing to body weight, mostly in pediatric patients (2). We have now entered an exciting era of medicine with major advances in the field of system biology and there is a realistic perspective that patients with tuberculosis will substantially benefit from these developments (3–5).

**Figure 1:**
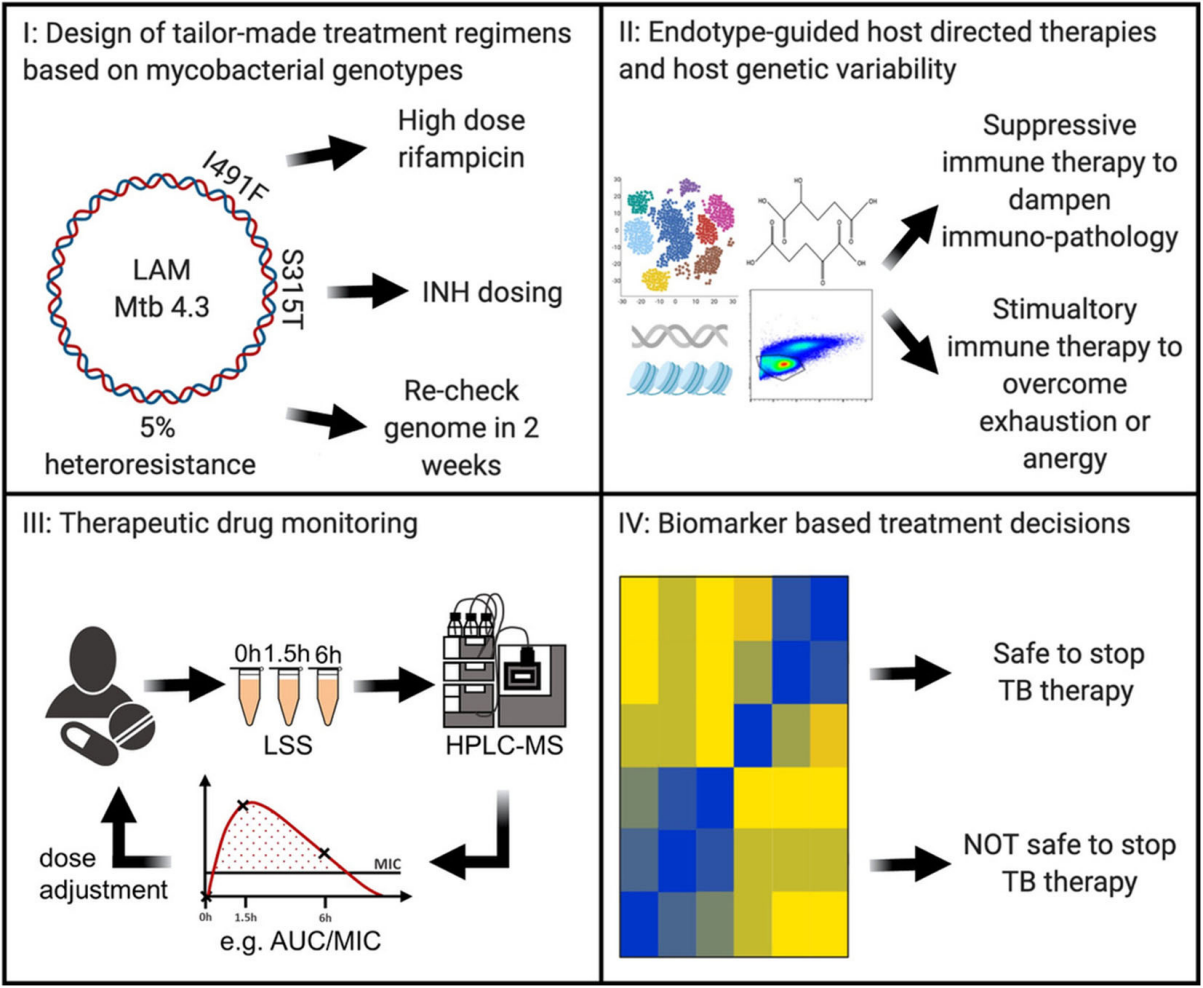
In the near future, Precision Medicine for tuberculosis will likely include (I) antibiotic regimens based on next-generation sequencing of the *Mycobacterium tuberculosis* genome to guide tailor-made therapies; (II) evaluation of gene expression, genetic, epigenetic, metabolism, and/ or immune phenotyping to discern the host endotype with endotype-specific host directed therapies to shorten and improve clinical outcomes; (III) individualization of antibiotic dosing through therapeutic drug monitoring; (IV) in treatment biomarker levels to customize therapy duration. LAM, lipoarabinomannan; Mtb, *Mycobacterium tuberculosis*; INH, isoniazid; LSS, limited sampling strategy; HPLC-MS, high-performance liquid chromatography-mass spectrometry; AUC, area under the curve; MIC, minimal inhibitory concentration; TB, tuberculosis.

In the future, tuberculosis patient care may be individualized in at least 4 areas (Figure 1): (I) next generation sequencing of microbial *M. tuberculosis* DNA can predict drug susceptibility in the first week of diagnosis (6); (II) host immunity can be detrimentally suppressed and need augmenting or it can be detrimentally exuberant and need to be suppressed (7–9); or other forms of host genetic variability that may be specifically addressed by immune-based interventions (10), (III) individualized drug concentrations that can guide antimicrobial dosing (11), and (IV) biomarkers that predict relapse-free cure and can guide duration of required therapeutics (12) (Figure 1). Such stratified therapies are within reach and could soon become available for the clinical management of tuberculosis.

*Precision Medicine* refers to prevention and treatment strategies that take individual variability into account and predict, who will benefit most from specific therapies and who will not. Taken together, Precision Medicine will improve patient outcome and reduce cost.

## Subsections Relevant for the Subject

### I: Design of Tailor-Made Treatment Regimens Based on Mycobacterial Genotypes

Drug resistances in *M. tuberculosis* complex strains are exclusively mediated by genomic variants, mainly single nucleotide polymorphisms (SNPs) and small insertions/deletion (indels). Acquisition of resistance genes via plasmids or horizontal gene transfer does not occur (13). Accordingly, resistant phenotypes of *M. tuberculosis* complex strains mainly have a clear genetic correlate, which means SNPs can be used for resistance predictions with very high precision and are expected to replace phenotypic drug susceptibility testing (DST) in the future (13). The virtually complete interrogation of all resistance associated variants in genomes of clinical *M. tuberculosis* complex strains has become possible by advances of next generation sequencing (NGS) techniques. NGS allows for rapid sequencing of full genomes or targeted sequencing of resistance genes (amplicon sequencing) even directly from patient specimens, i.e., sputum (14–16). While genomic variant detection is of very high accuracy, genotypic drug susceptibility testing performance is strongly dependent on the underlying knowledge database (17). Although there is no standardized and globally adopted, “resistance mutation catalog” available yet, recent studies demonstrate that genotypic DST can predict resistance to the first-line drugs isoniazid, rifampicin, pyrazinamide with more than 90% sensitivity and more than 95% specificity, and thus would meet the WHO target product profile for molecular DST assays (6, 18). However, resistance prediction for some drugs, such as ethambutol and pyrazinamide is problematic due to breakpoint artifacts in phenotypic DSTs (19) or gaps in current knowledge databases, i.e., yet unknown resistance mutations.

There is emerging evidence that genotypic DST has a very good prediction for phenotypic drug susceptibility (20, 21) although for some drugs like PAS, D-cycloserine, thioamides, linezolid, meropenem-clavulanate, bedaquiline, clofazimine, and delamanid data are still limited. Programmes aiming to fill the knowledge gap in “geno-to-pheno” prediction such as CRyPTIC (www.crypticproject.org) and ReSeqTB (platform.reseqtb.org) are currently collecting genome sequencing data of global strain collection in combination with phenotypic DST data to further improve genotypic resistance predictions. Here, sequencing approaches represent an effective alternative to rapidly generate comprehensive resistance profiles of multidrug-resistant (MDR) strains allowing the design of a successful MDR-TB therapy for around 90% of the MDR-TB patients (18, 20).

Genotypic DST methods already provide accurate predictions for first-line anti-tuberculosis drugs and guide the design of MDR-TB therapies (6, 20). Sputum sequencing together with an improved interpretation catalog of drug resistance defining mutations will potentially allow genotypic DST to replace phenotypic DST for a large fraction of clinical *M. tuberculosis* complex strains.

### II: Endotype-Guided Host Directed Therapies and Host Genetic Variability

Tuberculosis endotypes are the distinct molecular pathways through which an individual can progress to active tuberculosis. In a rough generalization, tuberculosis host immunity endotypes can be classified as immune deficient or as immune exuberant. The best described tuberculosis endotypes are deficiencies in interleukin (IL)-12, interferon (IFN)-γ or tumor necrosis factor (TNF) pathways, which result in decreased intracellular killing of *M. tuberculosis* (22, 23). However, mouse and human studies also demonstrated that exuberant IFN-γ or TNF is detrimental, resulting in pulmonary and macrophage necrosis and the escape of viable *M. tuberculosis* into extracellular space (8, 24–26). These studies demonstrate that a single immune correlate of protection is unlikely to be identified as the host immunity can not be either deficient or overly exuberant. Therefore, implementation of host-directed therapies (HDT) should preferably not be indiscriminate, but needs to be guided by the tuberculosis immune endotype. Preliminary work supports the identification of divergent host endotypes with contradictory metabolic, epigenetic, and immune gene expression endotypes (7), but these transcriptional studies lack corresponding functional studies.

In the pre-antibiotic era, treatment for tuberculosis was limited to host-directed therapy aiming at improving the nutritional and immune status of patients, e.g., with cod liver oil and sunlight (27), resulting in ~20% survival rate for a period of at least 10 years (28). Harnessing and targeting host immunity, while minimizing immunopathology is the critical next phase in improving anti-tuberculosis therapy. Implementing endotype-specific host-directed therapy, first require proper characterization of the distinct tuberculosis endotypes in order to identify amendable targets. Tuberculosis dampens host immune responsiveness (29–33), and therefore transcriptomic and immune functional studies must be implemented both at baseline and upon antigenic stimulation. Since 1927, we have known that metabolism drives immunity (34) and modern studies are defining the metabolic-epigenetic-immune axis (35–39). Therefore, ideally, transcriptomic, metabolic, epigenetic and functional immune studies would be paired with robust clinical information that would allow for multi-modal data integration between molecular data and clinical outcomes. Finally, an immune deficient vs. immune exuberant model, while helpful, is overly simplistic as single cell studies have demonstrated deficiency in one arm of immunity, while exuberance in another. For example, tuberculosis induced hemophagocytic lymphohistiocytosis (HLH) is characterized by exuberant monocytes and deficient cytotoxic lymphocytes (40–43).

Endotype characterization studies likely will need to occur at disease diagnosis and again weeks into therapy. Tuberculosis is the archetypical chronic infection, hence host immunity after months of chronic antigenic stimulation is unlikely to resemble host immunity at the beginning of infection. Upon initial antigenic stimulation, modulated by inositol triphosphate receptors, there is an influx of calcium that helps stimulate glycolysis and activates nuclear factor of activated T-cells (NFAT) to translocate into the nucleus (44) as an example. When immune activation is chronic, NFAT switches from heterodimerizing with activator protein (AP)-1, to forming homodimers that drive anergy and thymocyte selection-associated high mobility group box protein TOX (TOX) and nuclear receptor 4A (NR4A) and epigenetic-mediated immune exhaustion (45–50). Therefore, drugs that modulate calcineurin-NFAT, such as cyclosporin A or tacrolimus, could detrimentally inhibit host immunity early in the disease process by blocking AP-1-NFAT heterodimers that drive beneficial immunity. In contrast, late in the disease process, these same drugs may be beneficial by blocking the detrimental NFAT homodimers that drive anergic and immune exhaustion responses. Evaluating endotypes could be operationalized by tests such as a mycobacterial growth inhibition assay, a test that has identified ~50% of tuberculosis patients to have good mycobacterial killing capacity and ~50% to have deficient killing capacity (51), and an ELISA based test, such as a modified version of the QuantiFERON-TB Gold in-tube assay or T-SPOT.TB assay.

Host genetic studies have thus far largely focused on susceptibility to active tuberculosis (52, 53), to a lesser extent on susceptibility to infection (54) while the relationship between host genetic variability and treatment outcome is largely unexplored.

With regard to anti-tuberculous treatment, genetic studies have focused on drug exposure and toxicity (“pharmacogenetics”). The strongest association established is between N-acetyltransferase (NAT)2-acetylator status and isoniazid drug concentrations, microbiological failure, relapse (55), and hepatotoxicity (56) and probably, genetic variation underlies rifampicin bioavailability and aminoglycoside-induced hearing loss (10). A next step would be “Mendelian randomization” studies: associating genetic loci predicting drug concentrations to treatment outcome (rather than toxicity). Then, pharmacogenetic trials could be initiated in which patients are randomized to standard treatment vs. genotype-based drug dosing.

In the most extreme disease phenotypes, targeted HDT may be most relevant. A Leukotriene A4 hydrolase (LTA4H) promotor genotype, important for the balance between pro- and anti-inflammatory eicosanoids (24), predicted cerebrospinal fluid leukocyte count. The hyper-inflammatory genotype was associated with improved survival in HIV-negative patients receiving corticosteroids (57, 58). In contrast, the same LTA4H genotype did not predict mortality in another cohort of patients (59), possibly because of higher disease severity, but the conflicting results could also be driven by genetic factors such as shorter regions of linkage disequilibrium or plasticity in the direction of the effect of the polymorphism (60). This shows that host genetic studies should be replicated in different settings. This can result in randomized controlled trials with stratification according to genotype, as is currently done in a study among patients with tuberculous meningitis, where only those with the hypo-inflammatory LTA4H genotype are randomized to receive corticosteroids or placebo (and all with a hyperinflammatory genotype receive corticosteroids) (61).

Genetic studies can be strengthened by an integrated or “cross-omics” approach. Among patients with tuberculous meningitis, using unbiased metabolomics, it was found that higher cerebrospinal fluid tryptophan, which can affect *M. tuberculosis* growth and central nervous system inflammation, was associated with higher mortality. Using a genome-wide association approach, so-called quantitative trait loci (QTLs) that associate with cerebrospinal fluid tryptophan levels were identified, and those same QTLs predicted survival in 285 other patients (62). The relevance of tryptophan metabolism, its genetic regulation, and the possible implications HDT are now being examined in more than 2,000 tuberculous meningitis patients (https://grantome.com/grant/NIH/R01-AI145781-01). Other “omics” data that could be used in such an integrated approach are transcriptional data or proteomics.

Future genetic studies will also benefit from precise patient characterization and combination with *M. tuberculosis* genotype (63), in a genome-to-genome approach (64). Also, replication studies in different settings, including relevant covariates, are needed and promising leads need to be trailed in studies focusing on outcome to identify genetic variants important in treatment decisions.

### III: Therapeutic Drug Monitoring

Therapeutic drug monitoring (TDM) is the concept of individualizing drug dosing by measuring the drug concentration in a patient's serum/plasma and adjusting the dose accordingly.

Consequently, TDM is indicated in settings with a risk of low or high tuberculosis drug exposures: presence of altered drug absorption, distribution, metabolism, or excretion (e.g., renal insufficiency), comorbidities that may affect exposure to anti-tuberculosis drugs [HIV infection, diabetes mellitus (65)], drug-drug interactions, and in patients who are slow to respond or with relapsed tuberculosis (66).

Low anti-tuberculosis drug concentrations can lead to inefficient mycobacterial killing, treatment failure, relapse and selection of drug resistance (67), whereas high concentrations may increase the risk of adverse effects (68).

The TDM process comprises (1) obtaining blood samples, (2) measuring drug concentrations, and (3) interpreting the results.

1. The optimal sampling time points and the number of samples depend on the assessed drug. Limited sampling strategies (LSS) predict the most informative sampling time points based on population pharmacokinetic data, i.e., “average” exposures from tuberculosis patients. With the help of LSS, sampling can often be reduced to three or less time points (69, 70).
2. Analysis of the serum/plasma samples is ideally performed with high performance liquid chromatography mass spectrometry (HPLC-MS). HPLC-MS is highly sensitive and specific and allows simultaneous analysis of several tuberculosis drugs in one run of the assay (71).
3. The degree of exposure that is effective depends on the susceptibility of the patient's *M. tuberculosis* strain, as reflected in the minimal concentration that inhibits growth of *M. tuberculosis* (minimal inhibitory concentration—MIC). Dependent on the drug's mode of action, targets are defined as area under the curve (AUC)/MIC, maximal concentration (Cmax)/MIC, or time above MIC (T>MIC). Unfortunately, specific targets are not yet available for anti-tuberculosis drugs and MIC is seldomly determined. Alternatively, drug concentrations are compared to population pharmacokinetic data (66).

Linking NGS data for prediction of minimal inhibitory concentrations (MIC) with TDM data is a very promising concept to provide tailored high-dosage therapies in cases of low level bacillary drug resistance (21, 72). More clinical information will be needed to proof the plausible assumption that mutations in the genome of *M. tuberculosis* corresponding with a mildly elevated MICs to specific medicines can be overcome by a higher-dose administered of these drugs.

There is a disparity between tuberculosis prevalence and available resources for its treatment. TDM is highly resource-intensive with limited reimbursement available to offset cost. As health care providers avoid the costs of purchase and maintenance of equipment without reimbursement, very few centers have implemented TDM in their routine clinical practice. Ideally, results should be available and interpreted within days in order to effectively adjust therapies. New sampling techniques (73, 74), development of automated analytical techniques (immuno-assays) and *in-vitro* models that help to substantiate target values (75) will facilitate its roll-out. TDM integrates information on drug pharmacokinetics and mycobacterial susceptibility to ensure efficacy and prevent toxicity. It could improve the use of currently available drug therapy and individualized high-dose treatments may even overcome some forms of resistance.

### IV: Biomarker Based Treatment Decisions

An ideal treatment monitoring test would have a 1–2 day turn-around, be available and implementable in resource constrained settings and could accurately identify when tuberculosis therapy can be terminated to minimize excess treatment. Molecular tests such as the GeneXpert (Cepheid, USA) or the line-probe assays (Hain Life Sciences, Nehren, Germany) are commercially available as rapid diagnostic methods that permit drug-resistance prediction for important first- and second-line drugs (76, 77). As an alternative, transrenal DNA, and lipoarabinomannan (LAM) detection in urine have been described to diagnose active tuberculosis in people living with HIV (78–80). Screening for tuberculosis with a LAM urine assay in African people living with HIV and low CD4 count lead to a significant decrease in mortality (81). A novel assay with increased sensitivity may even have a larger impact for the management of HIV-associated tuberculosis (82). As an alternative, the detection of mycobacterial DNA from stool samples of patients with tuberculosis highly correlated with sputum-based diagnostic results and were also able to identify patients at risk for experiencing therapy failure (83).

Immunological assays could improve outcomes in populations in whom diagnosis is very difficult, such as children in whom reports of the T-cell activation marker (TAM-TB) test suggest a sensitivity and specificity of ~83 and 97%, respectively (84). Complex analysis of transcriptomic studies from whole blood have indicated RNA signatures to be associated with future active tuberculosis although the applicability of such tools in a low or middle income country context is uncertain (85–87). However, a recent systematic review and patient-level pooled meta-analysis concluded that blood transcriptional biomarkers reflect only short-term risk of active tuberculosis and surpass WHO benchmarks only if applied to 3–6-month intervals (88).

In addition, certain computer-aided diagnostic (CAD) tools may be able to identify tuberculosis patients with high accuracy (specificity of 98% and of sensitivity 90%) from digital chest-X-ray images (89).

Mycobacterial culture is the most relevant measure of treatment response, but mycobacterial growth is slow and, as treatment progresses, the time to a positive result increases (90). Here, rapid molecular tests detecting only viable bacteria such as the molecular bacterial load assay (MBLA) may be promising for the future treatment monitoring of tuberculosis patients (91). The MBLA correlates with the time to liquid culture positivity. The advantage is that it is rapid and not compromised by contamination of culture. MBLA test results correlates with disease severity, and provide information on the bactericidal effect of different drugs and drug regimens (91–93). As a rapid test measuring the number of viable organisms in a few hours it has potential to identify failing patients. This could suggest infection with a resistant organism or non-adherence and enable additional investigations or alternatively provide reassurance that the patient is responding appropriately. Operational trials to explore this are now underway.

Phenotypic changes on *M. tuberculosis*-specific blood T cells may be able to inform about treatment efficacy as shown in adults and in children (94, 95). Modern imaging techniques such as PET-CT scans may correlate with treatment responses, but alone were not accurate enough to precisely identify patients with recurrent disease in South Africa (96). Interestingly, certain RNA signatures could predict recurrent disease in tuberculosis patients (97, 98). However, these biomarkers have not been prospectively evaluated and markers that could individualize the duration of therapy are missing so far.

## Discussion

With technological advances in the field of diagnostics, analytics, and integration of comprehensive data-sets, tailor-made Precision Medicine for patients with tuberculosis is within reach at centers that operate on the frontier of translational research (Table 1). As independent measures, genotypic prediction of phenotypic *M. tuberculosis* drug-resistance based on information of entire bacterial genomes, genotypic and phenotypic identification of immune endotypes and human susceptibility to tuberculosis to individualize HDT, and novel biomarkers guiding physicians for individual treatment decisions are already in place at highly specialized centers and usually under research conditions. The clinical application of some of these innovations needs to become the medical standard. However, as a poverty-related disease, the majority of patients affected by tuberculosis live in resource limited settings. Finding funding and performing operational research on the implementation of precision medicine for tuberculosis in these settings will be one of the great challenges for the future.

**Table 1:** Current standards and future perspectives for Precision Medicine for tuberculosis.

**Measure**	**Current standard**	**Future perspective for precision medicine**	**When to apply**
Rapid selection of effective anti-tuberculosis drugs to form a treatment regimen.	Rapid molecular Rifampicin-resistance testing followed by phenotypic drug susceptibility testing in liquid and/or solid media cultures.	Rapid, sputum-based automated sequencing of the entire genome of *M. tuberculosis* or of genetic regions (amplicons) of the genome of *M. tuberculosis* where mutations do predict drug resistance. Algorithm-based treatment decisions based on molecular prediction of drug resistance.	At the time of diagnosis.
Supporting the host immunity by endotype-guided decisions for host-directed therapies.	Endotypes of tuberculosis are still not well defined and identification of endotypes to guide host directed therapies is not performed at present.	Optimal testing needs to be discerned, but likely will include a mixture of metabolism, genetic, epigenetic and/or immune functional studies to identify the host endotype. For example, if host immunity was found to be exuberant, then an endotype-specific therapy might consist of a glucocorticoid, NSAID, calcineurin inhibitor (cyclosporin or tacrolimus) or mTOR inhibitor (rapamycin). In contrast, if evaluations identified anergic or exhausted immunity, than immune boosting regimens may be chosen.	Within the first week: of the diagnosis. Should be repeated 4–8 weeks to evaluate dynamic transitions.
Analysis of host genetic variability to predict adverse events and to provide precise therapeutically interventions.	Host genetic markers are currently not identified in clinical practice. At specialized centers and on special request genetic markers such as mutations associated with specific immune deficiencies are evaluated.	Genetic testing before the start of treatment to (1) define dosing of anti-tuberculous treatment and to (2) identify patients susceptible to adverse drug events and (3) to tailor host-directed therapy to the individual patient.	At the time of diagnosis.
Therapeutic drug monitoring.	Only very few centers world-wide perform measurement of drug levels and PK/PD profiles of anti-tuberculosis drugs in routine clinical practice. Even at these centers there are no analytic capacities to monitor several of the 2nd-line anti-tuberculosis drugs.	Regular therapeutic drug monitoring with same-day-results for all anti-tuberculosis drugs for individual dosage adjustments.	For the first month once a week, once a month thereafter throughout the course of treatment.
Individualizing the duration of anti-tuberculosis therapy.	There are no biomarkers available for routine clinical practice to guide clinicians in the decision of the duration of anti-tuberculosis therapy.	Defining the duration of therapy to achieve relapse-free cure based on the measurement of a robust validated biomarker that also identifies patients having the risk for experiencing recurrent disease at early time points during therapy. Ideally, this biomarker should be measurable in a point-of-care test system.	Once a month throughout the course of treatment starting at month 4.

## Author Contributions

All authors made a contribution to the acquisition of the information for the work, critically revised the manuscript for important intellectual content, and gave final approval of the current version to be published. All authors agree to be accountable for all aspects of the work in ensuring that questions related to the accuracy or integrity of any part of the work are appropriately investigated and resolved.

## Conflict of Interest

JH, MR, and CL have filed a patent for a 22-gene model to predict the end of therapy of TB treatment (EP20158652.6). The remaining authors declare that the research was conducted in the absence of any commercial or financial relationships that could be construed as a potential conflict of interest.
